# Atherosclerotic Plaque Biomarkers: Beyond the Horizon of the Vulnerable Plaque

**DOI:** 10.2174/157340311795677680

**Published:** 2011-02

**Authors:** Guus W. van Lammeren, Frans L. Moll, Gert Jan De Borst, Dominique P.V de Kleijn, Jean-Paul P.M. de Vries, Gerard Pasterkamp

**Affiliations:** 1 Experimental Cardiology Laboratory, University Medical Center Utrecht, The Netherlands; 2 Department of Vascular Surgery, University Medical Center Utrecht, The Netherlands; 3 Department of Vascular Surgery, St Antonius Hospital Nieuwegein, The Netherlands

**Keywords:** Atherosclerosis, biomarkers, cardiovascular outcome, prediction, vulnerable plaque.

## Abstract

Cardiovascular disease (CVD) is the number one cause of death globally, and the majority of CVD is caused by atherosclerosis. Atherosclerosis is a systemic inflammatory disease that leads to myocardial infarction, stroke and lower limb ischemia. Pathological studies have given insight to development of atherosclerosis and the importance of local plaque vulnerability, leading to thrombus formation and cardiovascular events. Due to the burden of cardiovascular disease, identification of patients at risk for cardiovascular events and treatment stratification is needed. The predictive power of classical risk factors is limited, especially in patients with manifest atherosclerosis. Imaging modalities have focused on the characteristics of the vulnerable plaque. However, it has become evident that not all so-called vulnerable plaques lead to rupture and subsequent thrombosis. The latter obviously limits the positive predictive value for imaging assessment of plaques and patients at risk. Serum biomarkers have also been studied extensively, but have very limited application in a clinical setting for risk stratification. In line with the important relation between vulnerable plaques and cardiovascular events, plaque biomarker studies have been initiated. These longitudinal studies are based on the concept, that a vulnerable plaque contains predictive information for future cardiovascular events, also in other territories of the vascular tree. Results look promising and plaque markers can be used to develop imaging modalities to identify patients at risk, or to monitor treatment effect. Plaque biomarker studies do not challenge the definition of the vulnerable plaque, but use its concept in favor of prediction improvement for vascular patients.

## INTRODUCTION

Cardiovascular disease (CVD) is the major cause of morbidity and mortality worldwide and is predominantly caused by atherosclerosis [1]. Atherosclerosis is an inflammatory process resulting in local plaque deposition in the vessel wall of arteries, leading to luminal narrowing and decreased tissue perfusion. Although atherosclerotic plaque development is a local process in the vessel wall that can give rise to symptoms in one specific area, it is also a systemic condition with simultaneous plaque formation in different areas of the vascular tree [2, 3]. Patients can present with myocardial ischemia if coronary arteries are affected; with cerebrovascular events if carotid arteries are affected or with lower limb ischemia if peripheral arteries are involved. The systemic character of atherosclerosis is one of the key, and disadvantageous properties of the disease. Classical risk factors for development of CVD have been identified from population based studies and include age, gender, cigarette smoking, high blood pressure, elevated cholesterol levels, diabetes mellitus, obesity and familial history of coronary heart disease [4-8].

## DEVELOPMENT OF ATHEROSCLEROSIS

The important role of lipids in the development of atherosclerosis was described by Ross in 1976 [9]. In addition to hyperlipidemia, the role of inflammation in atherosclerotic plaque formation and progression is yet undisputable [10, 11]. In short, initiation of atherosclerosis starts with an increased permeability of the arterial endothelium, which facilitates migration of cholesterol-filled low density lipoprotein (LDL) particles into the vessel wall. After migration into the intimal layer, LDL particles become oxidized. This results in an inflammatory response and the attraction of monocytes to the lesion. Monocytes differentiate into macrophages and take up the oxidized lipoproteins and turn into foam cells. Foam cells eventually precipitate in the vessel wall causing a fatty streak, which further stimulates the inflammatory process. Further attraction of macrophages is promoted, together with migration of proliferating smooth muscle cells (SMC) from the medial into the intimal layer of the arterial wall. SMCs produce collagen which results in formation of a fibrous cap overlying the atheroma; and covering the atherosclerotic plaque.

## THE VULNERABLE PLAQUE

Postmortem studies revealed that acute myocardial infarction and coronary death were associated with coronary plaque disruption caused by fibrous cap rupture, superimposed thrombus formation and coronary occlusion. Thinning of the fibrous cap was shown to be caused by a decreased collagen production as a result of decreased SMC content, or degradation by matrix metalloproteinases (MMPs) excreted by macrophages [12]. This led to the description of the entity vulnerable plaques by Davies in 1996, who stated that plaques with large lipid cores, low SMC content, high macrophage content and a thin fibrous cap are rupture prone and therefore vulnerable [13]. Based on these principles and plaques found in patients who died from sudden coronary death, a morphological classification and scoring system for atherosclerotic lesions was established by Virmani *et al.* [14]. In 2003, the importance of intraplaque hemorrhage for progression of atheroma was elucidated by Kolodgie *et al.* [15]. The researchers showed that intraplaque hemorrhage contributes to deposition of free cholesterol, macrophage infiltration and enlargement of the necrotic core which increases the risk of plaque destabilization. One of the causes for intraplaque bleeding was hypothesized to be related to leaky microvessels inside the atherosclerotic plaque. Intraplaque microvessels result from angiogenesis, most likely under influence of hypoxic and inflammatory stimuli [16]. The leaky vessel hypothesis was recently extended by Sluimer *et al.* who showed that increased microvessel density was associated with an unstable plaque morphology and rupture prone plaques [17]. Compromised integrity of the endothelium of microvessels was held responsible for leakage of red blood cells into the atherosclerotic plaque, giving rise to intraplaque hemorrhage. A schematic illustration of the current view on stable and unstable atherosclerotic plaques is depicted in Fig. (**1**). The vulnerable plaque concept also applies for carotid plaques. Vulnerable plaques were observed in symptomatic patients, who suffered from cerebrovascular events like stroke and transient ischemic attack (TIA), as opposed to asymptomatic patients, who had relatively stable plaques [18]. These findings confirmed that local plaque processes are associated with local plaque stability and the occurrence of an event in the respective area of the vascular tree.

## THE SEARCH FOR BIOMARKERS TO DETECT THE VULNERABLE PATIENT

Based on classical cardiovascular risk factors, the Framingham Heart registry identified clinical predictors for adverse cardiovascular outcome in the entire population [19]. Within the domain of patients with vascular disease, classical risk factors have limited discriminative values, because most patients have a certain amount of the risk factors. Due to the growing evidence of the association of vulnerable plaques with cardiovascular events, a consensus statement was published in 2003 by Naghavi *et al.* to encourage translational research for the identification and treatment of vulnerable patients, based on the concept of the vulnerable plaque [20]. Taking the concept of the vulnerable plaque as a starting point, a more systemic approach was considered to identify patients at risk for cardiovascular death. This resulted in numerous studies in search of so-called biomarkers. A biomarker is defined as a characteristic that is objectively measured and evaluated as an indicator of normal biologic processes, pathogenic processes, or pharmacologic responses to a therapeutic intervention [21]. Due to the pivotal role of lipids and inflammation in development of atherosclerosis, cholesterol and inflammatory markers were thoroughly studied. The predictive value of cholesterol markers for cardiovascular events is limited, because more than half of all vascular events occur in individuals with normal cholesterol levels [22]. Lipoprotein-associated phospholipase A2 (Lp-PLA2) has been described as a potential marker for CVD and mortality [23]. However, Lp-PLA2 levels drop significantly after stroke and myocardial infarction [24]. As a result of this unstable marker property, the use of Lp-PLA2 might be less reliable for long-term risk stratification shortly after primary events.

The inflammatory biomarker c-reactive protein (CRP) has been reported to be a predictive marker for cardiovascular events among healthy individuals; and that it adds prognostic information to the Framingham risk score [22]. The JUPITER trial investigators concluded that high-sensitivity CRP and LDL levels in healthy individuals can be lowered by prophylactic treatment with statins, which also resulted in a significant decrease of cardiovascular events, even in the absence of hyperlipidemia [25, 26]. This indicates that CRP can be used to identify patients at risk; and that CRP and LDL can be used as biomarkers for treatment effect. Nevertheless, an inflammatory marker like CRP has its limitations, since it is not specific for atherosclerosis and cardiovascular events. Serum CRP levels dramatically increase in case of any infection or tissue damage caused by trauma [27] and it is therefore controversial to start aggressive treatment solely based on elevated CRP levels. Recent analysis of inflammatory markers, including hs-CRP, interleukins 6, 10 and 18, soluble CD40 ligand, P- and E-selectin, NT-proBNP, fibrinogen and cystatin C, in patients with acute coronary syndrome showed that all markers by themselves offer only limited incremental information to clinical risk scores [28]. However, a combination of fibrinogen and NT-proBNP contained predictive information in addition to clinical parameters.

Besides serum marker studies, non invasive imaging modalities have been studied extensively. Imaging can be considered useful for screening for vulnerable plaques and thereby identifying high risk patients, but also to monitor the stabilizing effect of treatment on local plaque composition. Imaging of plaque inflammation has been extensively studied with positron emission tomography (PET), and has shown its potential since 2002 [29]. Imaging of proteolytic enzymes such as MMPs is of interest, since these proteases are involved in degradation of the fibrous cap of the plaque, but are also predictive for future events [30, 31]. Recently it was shown, that labeled MMP-2 and MMP-9 can be imaged in human carotid plaques, but in an *ex vivo* situation [32]. In spite of ongoing improvements for plaque imaging, also limitations are present. Not all vulnerable plaques, also called thin fibrous cap atheroma (TFCA) are likely to progress to rupture and cause cardiovascular events [33]. This limits the positive predictive value of plaque imaging for cardiovascular events and identifying the patient at risk. Consequently, a clinical decision for invasive treatment of a TFCA lesion detected with imaging, cannot be made on these bases. It is therefore important to further define other markers of instability and indicators plaque rupture. Besides limitations in positive predictive power, also technical improvements for imaging modalities like PET scans are warranted, especially for the contrast difference between the lesion and surrounding background (target-to-background ratio) and limited spatial resolution (partial volume effect) [34]. Secondly, the use of radioactive material and high costs of a PET scan are clearly limitations of this diagnostic tool.

**Fig. (2):**
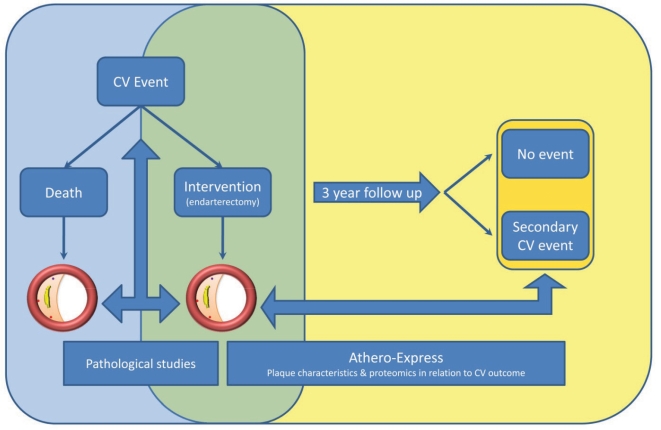
Concept of pathological studies and longitudinal translational biobank studies with the atherosclerotic plaque as a starting point. In blue, the design of postmortem and histopathological studies allowing crossectional research to investigate the association of plaque type with the initial event. In green the overlap between crossectional and prospective biobank stusdies. In yellow the unique Athero-Express study with a longitudinal design, with the ability to investigate the association of local plaque characteristics with systemic cardiovascular outcome.

Combining the limitations of serum markers and imaging studies, it is clear that there is a need for more specific and sensitive predictors for primary cardiovascular events, but also for secondary events in vascular patients at risk. Based on these needs and the central role of the vulnerable plaque in the occurrence of (cardio)vascular events, the Athero-Express study was designed [35]. The study design is based on local plaque information and the fact that atherosclerosis is a systemic condition. Atherosclerotic plaque formation occurs throughout all large arteries in the human body, which might imply that local plaque properties from one plaque in the vascular tree might be extrapolated to other atherosclerotic lesions at a distant location. These specific properties could reveal information regarding the stability of not just the local plaque, but the stability of the entire atherosclerotic process within an individual. In Athero-Express, human atherosclerotic plaques are harvested during carotid and femoral endarterectomy. Plaques are analyzed histologically and proteins are isolated to measure inflammatory interleukins, MMPs and other inflammatory markers. All patients are prospectively followed for three years after vascular surgery and endpoints are registered. The Athero-Express study design facilitates research that relates plaque properties to cardiovascular events during follow up of vascular patients (Fig. **2**). 

## RESULTS FROM LONGITUDINAL PLAQUE BIOMARKER STUDIES

The carotid plaque contains valuable information for follow up after vascular surgery. Hellings *et al.* showed that local plaque characteristics are associated with restenosis at the site of carotid endarterectomy after 1 year [36]. Endarterectomy of lipid-rich, inflammatory plaques was associated with reduced risk of restenosis compared to stable, fibrous plaques, independent from clinical characteristics.

Recently Hellings *et al.* concluded that carotid plaque composition also contains predictive information for future cardiovascular events elsewhere in the vascular system, independent from established risk factors and medication use [37]. Patients undergoing carotid endarterectomy, with a local plaque containing intraplaque hemorrhage or marked intraplaque microvessel formation demonstrated an increased risk of secondary cardiovascular events with Hazard Ratios of 1.7 [1.2-2.5] and HR=1.4 [1.1-1.9], respectively. Other histological parameters like macrophage infiltration large lipid core, calcifications, collagen and smooth muscle cell infiltration were not associated with secondary events.

On protein level, De Kleijn *et al.* demonstrated that carotid plaque osteopontin (OPN) level is a predictor of cardiovascular events after carotid endarterectomy [38]. Patients with plaque OPN levels in the upper quartile had a nearly 4-fold risk for development of cardiovascular events compared with patients with OPN levels in the lowest quartile (adjusted HR = 3.5 [2.0-5.9]). 

## LIMITATIONS AND FUTURE PERSPECTIVES

Plaque biobank studies with a longitudinal design, focusing on predictive plaque biomarkers clearly do not challenge the definition of the vulnerable plaque, but use information gathered from pathological studies for prediction of cardiovascular events in a more systemic manner as opposed to pathological studies focusing on local plaque characteristics and local outcome. In addition, local plaque biomarkers that are predictive for future events, can give rise to the use of imaging techniques to identify and monitor patients at risk. Due to the important role of microvessels and intraplaque hemorrhage for development of vulnerable plaques, but also the predictive value for future events, imaging modalities for these plaque characteristics are of great interest [39]. A prospective MRI study of carotid plaques showed that intraplaque hemorrhage at baseline was associated with plaque progression and increasing lipid core volume over an 18 month period [40]. Carotid plaque hemorrhage detected on MRI in symptomatic patients was associated with embolization during carotid endarterectomy, which might result in a higher periprocedural risk, but this has to be confirmed in larger studies [41]. Also in patients with only moderate carotid stenosis (30-69%), MRI detected intraplaque hemorrhage was associated with ipsilateral ischemic events during an 28 month follow up period [42]. But also this study included only a limited amount of patients, therefore larger series will have to be obtained. Also the presence of intraplaque hemorrhage in asymptomatic patients and the association with ipsilateral ischemic events will have to be studied.

Plaque biobank studies also have certain limitations. Endarterectomy is an invasive surgical procedure. Carotid or femoral plaque analysis is therefore limited to patients who undergo vascular surgery, which clearly limits the domain of patients suitable for risk assessment. Predictive plaque biomarkers therefore also require external validation and investigation of the predictive power in other populations. Nevertheless, findings from plaque biomarker research can be used for future imaging techniques as stated above. 

Furthermore the plaque analysis takes place at one time point, while it is known that atherosclerotic plaque composition changes over time. Peeters *et al*. showed that carotid atherosclerotic plaques stabilize over time after stroke [43]. Plaques from patients operated more than 30 days after the cerebrovascular event contained significantly less macrophages and interleukin 8 and 6. For the interpretation of plaque biomarkers, this time period has to be taken into account. 

## CONCLUSION

Atherosclerotic plaque biomarker studies show great potential. Future research for validation and application in imaging techniques is warranted. Plaque biomarker studies do not challenge the definition of the vulnerable plaque, but use its concept in favor of prediction improvement for vascular patients.

## Figures and Tables

**Fig. (1):**
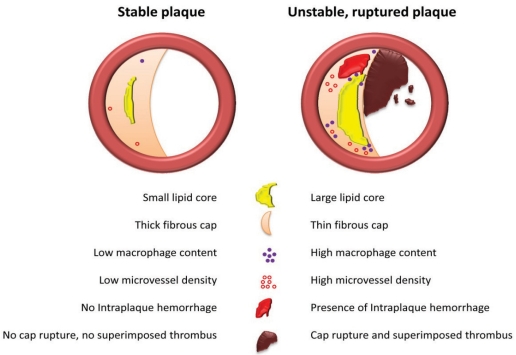
Schematic overview of a stable atherosclerotic plaque (left) and an unstable atherosclerotic plaque (right).
